# Canadian Senate Report on Obesity: Focusing on Individual Behaviours versus Social Determinants of Health May Promote Weight Stigma

**DOI:** 10.1155/2018/8645694

**Published:** 2018-07-02

**Authors:** Angela S. Alberga, Lindsay McLaren, Shelly Russell-Mayhew, Kristin M. von Ranson

**Affiliations:** ^1^Department of Exercise Science, Concordia University, 7141 Sherbrooke Street West Office: SP-165.31, Montreal, QC, Canada H4B 1R6; ^2^Community Health Sciences, Cumming School of Medicine, University of Calgary, TRW Building, 3rd Floor, 3280 Hospital Drive NW, Calgary, AB, Canada T2N 4Z6; ^3^Werklund School of Education, University of Calgary, 2500 University Dr. NW, Education Tower 634, Calgary, AB, Canada T2N 1N4; ^4^Department of Psychology, University of Calgary, 2500 University Drive NW, Calgary, AB, Canada T2N 1N4

## Abstract

Very little attention has been given to unintended consequences of government reporting on obesity. This paper argues that the 2016 Senate report, “Obesity in Canada: A Whole-Of-Society Approach,” exemplifies the systemic public health issue of weight stigma. The purpose of this viewpoint is to critique the approach taken in the Report, by illustrating that it (1) takes a weight-centric approach to health, (2) does not acknowledge important limitations of the definition and measurement of obesity, (3) reifies obesity as a categorical phenomenon that must be prevented, and (4) uses aggressive framing and disrespectful terminology. The Report perpetuates a focus on the individual, thereby failing to recognize the role that governments can play in reducing weight stigma and addressing social determinants of health. If steps are taken to avoid propagating weight stigma, future reports could more constructively address health promotion, equity, and social determinants of health in their policies.

## 1. The Senate Report on Obesity, 2016

In March 2016, the Standing Senate Committee on Social Affairs, Science and Technology of the federal government of Canada released a report entitled, “Obesity in Canada: A Whole-Of-Society Approach for a Healthier Canada” (“the Report”) [1]. The Standing Committee had been tasked with examining the causes and consequences of obesity and identifying how to address obesity in Canada. The Report was developed through meetings with Canadian and international stakeholders (e.g., researchers, advocates, medical experts, and civil servants) over the course of more than a year. The Report summarizes the prevalence, mortality rates, and healthcare costs of obesity in Canada; it discusses four main topics (food consumption trends, specific elements of diet, processed food industry, and daily lifestyles of Canadians), and it concludes with a list of 21 recommendations to address rising rates of obesity in Canada. On March 1, 2016, the government issued a news release with the headline “Urgent action needed to fight rising obesity rates in Canada says ground-breaking Senate Report” that garnered national media attention.

Our purpose is to detail important concerns with the content and tone of the Report, which we argue are emblematic of the weight stigma that pervades Canadian society. We argue that the Report perpetuates weight stigma by (i) using a weight-centric approach to health, (ii) not acknowledging important limitations of the definition and measurement of obesity, (iii) reifying obesity as a categorical phenomenon that must be prevented, and (iv) using aggressive framing and disrespectful terminology. We conclude with recommendations, including how governments in Canada and other countries can work to reduce weight stigma by addressing social determinants of health.

## 2. The Senate Report on Obesity: A Critique

Weight stigma, weight bias, weight prejudice, weight discrimination, body shaming, and antifat discrimination are synonymous terms used to describe negative attitudes, beliefs, and behaviours towards individuals who have been classified as overweight or living with obesity. Weight stigma can be implicit (i.e., unconscious), explicit (i.e., conscious), and/or internalized (i.e., belief that the stigma is deserved) and can be enacted in many forms, including through verbal, physical, or relational victimization and unequal treatment [2].

Weight stigma is fundamentally about equity, which may be defined as differences between social groups (e.g., those classified as with versus without obesity) that are deemed to be unfair and avoidable [3]. Evidence shows that people living with obesity are treated differently from people not living with obesity in numerous societal sectors [4]. Specifically, weight stigma has been shown to be pervasive in employment, healthcare, educational settings, and interpersonal relationships within families, friends, and intimate partners [5, 6]. It is linked to adverse mental, physical, and social health consequences [7] including disordered eating patterns, avoidance of social and physical activities, increased anxiety, depression, stress, and weight gain [4, 8]. Two recent studies suggested that the *stigma* associated with body weight, rather than weight itself, may be responsible for adverse health consequences [9], including increased mortality risk [7].

Guthman highlighted the epistemic construction of the “obesity epidemic,” which tends to neglect the felt experiences of those who are living with obesity and who feel stigmatized by how obesity is reported [10]. In line with Guthman's perspective, we argue that the Report is highly problematic in that it is centered in individually focused, uncritical obesity discourses that focus on individual choice and agency. The following four arguments demonstrate that the Report is emblematic of the weight stigma that pervades Canadian society.

### 2.1. The Report Takes a Weight-Centric Approach to Health

The World Health Organization defines health as “a state of complete physical, mental and social well being and not merely the absence of disease or infirmity” [11]. In contrast, the Report strongly focuses on a view of health as the absence of disease (i.e., obesity).

Although the WHO's definition conveys that there is more to health than just physical dimensions, the prominent focus of the Report is body weight. The Report does not provide recommendations to improve the mental and social well being of Canadians. The statement, “This report urges the federal government to take aggressive measures to return Canadians to healthy weights” ([1], p. 5) implies that Canadians were once at “healthy weights” and that there is a “healthy weight” for all Canadians, despite a lack of evidence supporting this claim. The Report equates weight with health and thereby conflates size and pathology [10]. Accordingly, the Report contributes to what O'Reilly and Sixsmith have described as a weight-centered, healthist, and moralizing “obesity” discourse likely to cause harm via weight cycling, eating disorders, mental health issues, and social stigmatization [12].

Further, one of the subheadings, “Tipping the Scales Towards a Healthy Future,” ([1], p. iv) and the consistent use of the phrase “healthy weights” throughout the Report highlight the idea that weight loss and the prevention of weight gain will improve the health of Canadians. However, there is some research that suggests that there are side effects of weight cycling and weight loss that do not necessarily improve some dimensions of health nor decrease the overall risk of acquiring other chronic diseases [13, 14].

Despite the Report's problematic focus on “healthy weights,” we acknowledge that the Report attempts to disconnect weight and health by suggesting a positive reframing of physical activity *not* as a strong component of weight management, but rather as important for overall health. The Report states “most witnesses agreed that physical activity itself may not be primarily to blame for the increase in obesity. However, they noted that physical activity can help to mitigate the negative health effects of excess body fat. As such, witnesses urged increased physical activity not as a means of weight loss but as a means of improving health outcomes.” ([1], p. 8). However, the Report's focus on diet and exercise also raises important concerns. That is, this focus on individual agency to pursue healthy behaviours such as healthy eating and physical activity bypasses addressing fundamental deeply rooted social determinants of health such as income, education, employment, early childhood development, stress, food insecurity, housing, that could influence the opportunity, ability, and inclination to partake in healthy behaviours [15–17]. We address this concern in more detail in Section 3.1.

### 2.2. The Report Does Not Acknowledge Limitations to the Definition and Measurement of Obesity

The World Health Organization (WHO) defines overweight and obesity as “abnormal or excessive fat accumulation that presents a risk to health” [18]. Guthman identified that this WHO definition of obesity, and the measurement of obesity through BMI, lacks clear definitions of “abnormal,” “excessive,” and “impaired” and does not explain how fat accumulation can impair health [10]. Though BMI is measured on a continuous scale whereby a person is classified as having obesity if their measured BMI is above 30 kg/m^2^ in adults and above the 95th percentile for children and teenagers [18], these cut-points, while convenient, are at least somewhat arbitrary. Furthermore, there are important limitations of BMI. For example, BMI has been widely criticized because it does not consider individual attributes that are known disease risk factors nor more importantly other social determinants of health known to influence health risk such as income, education, employment, early childhood development, stress, food insecurity, housing, and so on [15–17]. In light of these limitations, some have called for a revised definition of obesity [19] such as the Edmonton Obesity Staging System (EOSS). The EOSS classifies an individual's health risk using a 5-point ordinal scale, ranging from stage 0 (no risk) to stage 4 (end stage), based on weight-related comorbidities or weight management barriers (metabolic, mechanical, and mental health), to better identify individuals who may be at risk for diabetes, cardiovascular disease, and premature death. An analysis of the National Health and Nutrition Examination Surveys (NHANES) 1988–1994 and 1999–2004 showed that EOSS was a stronger predictor of mortality than BMI [20]. More epidemiological data analysis from the Aerobics Center Longitudinal Study showed that individuals with obesity who have EOSS stage 0 (zero risk) or stage 1 (mild risk) did not have an elevated mortality risk [21].

The Report accurately acknowledges that BMI may not be useful at the individual level by stating “The committee was told that while BMI is an appropriate measure for population-based studies, individual obesity may be better measured by waist circumference,” ([1], p.14). Despite that acknowledgement, the Report does not discuss these operational concerns nor recent debates about definitions and measurement of obesity [22]. For example, the Report does not acknowledge that waist circumference does not measure total body composition; rather, it measures only abdominal adiposity, and not visceral adiposity, which is associated with a higher metabolic risk. It has been suggested in more recent research that waist-to-height ratio may be a better anthropometric measure than BMI or waist circumference [23, 24].

Although suggestions for future definitions and measurement tools for obesity are outside the scope of this critique, it would have been helpful if the Report acknowledged the current debates on the meaning and value of BMI [22], acknowledged the debates on the relationship between BMI and mortality risk [25–27], discussed the limitations of BMI, and highlighted the need for more research in the diagnosis and measurement of obesity. Furthermore, it would have been appreciated if the Report highlighted the importance of measuring people's bodies sensitively by ensuring respect towards patients and preserving their dignity when weighing or measuring their bodies.

### 2.3. The Report Frames Obesity as Something That Must Be Prevented

The Report uses a disease prevention lens to achieve a “Healthier Canada” by focusing on altering behaviours that could lead to obesity. While disease prevention is a core function of public health [28] and, in light of growing costs of acute care, many would argue for the need for greater attention to prevention, the framing of obesity as something to be avoided is problematic.

First, the Report does not acknowledge potential drawbacks of current approaches to obesity prevention. Evidence suggests that certain policies for obesity prevention (e.g., BMI report cards in schools) may do more harm than good by focusing on weight as the best or only indicator of health [29–31]. Some obesity prevention campaigns are stigmatizing and have the opposite of the intended effect: they reduce self-efficacy and do not motivate positive behavioral changes [32–34]. Obesity prevention campaigns that stigmatize certain bodies may perpetuate stereotypes associated with weight and unsubstantiated judgments of people's characteristics, skills, and personalities based solely on weight status. It is also important to note that a focus on preventing obesity ignores the fact that people of all sizes can benefit from improved health behaviors. That is, healthy eating and physical activity should be promoted for health, and a focus on preventing obesity distracts from that goal.

Second, the Report's framing neglects individuals *living* with obesity; in particular, what it is like to have a body that is seen by powerful institutions (e.g., medicine and public health) as something to be prevented. Focusing public health efforts on the prevention of obesity overlooks the 5.3 million Canadian adults who are living with obesity [35] as well as the people categorized at normal, overweight, or underweight who do not practice healthy eating and exercise behaviors. The Report thereby neglects the importance of promoting health to individuals of *all* weights and sizes [36]. Although many credible experts were consulted as part of this report, people actually *living* with obesity were not consulted prior to its public release [37].

### 2.4. Aggressive Framing and Disrespectful Terminology

The Report discusses obesity with an aggressive and alarmist tone. For example, the Report states, “This report urges the federal government to take aggressive measures to return Canadians to healthy weights” ([1], p. v), “[T]he increased incidence of obesity among children is particularly alarming” (p. 14), “obesity crisis” (p. iv), “beat back this crisis” (p. iv), “this disturbing trend” (p. 1), “to combat obesity” (p. v), and “to fight obesity” (p. 22) [1]. The “Report Highlights” state, “There is an obesity crisis in this country. Canadians are paying for it with their wallets—and with their lives.” Puhl and colleagues have shown that the way we talk about obesity (i.e., the words we use) can reinforce stigma and can have the effect of decreasing motivation to promote behavior change [34]. The general alarmist tone of this report tends to evoke fear, blame, and shame related to obesity that could perpetuate weight stigma.

The Report consistently uses condition-first terminology, such as “obese adults” (p. 1), “obese children” (p. 1), “obese Canadians” (p. 2), and “obese women” (p. 18) [1], which does not align with recommendations outlined by professional organizations devoted to the prevention, management, and treatment of obesity in Canada and the United States. The Canadian Obesity Network, the Obesity Society (U.S.), and the Obesity Action Coalition (U.S.) recommend the use of person-first language in all reporting about obesity. Although differing opinions exist on preferred terminology when referring to “a person with obesity” or “people of size,” included by various other groups including feminists, fat activists, and critical obesity scholars [38], we suspect that the terminology used in this report would be viewed as pejorative by most professional organizations and various activist groups.

**Figure 1:**
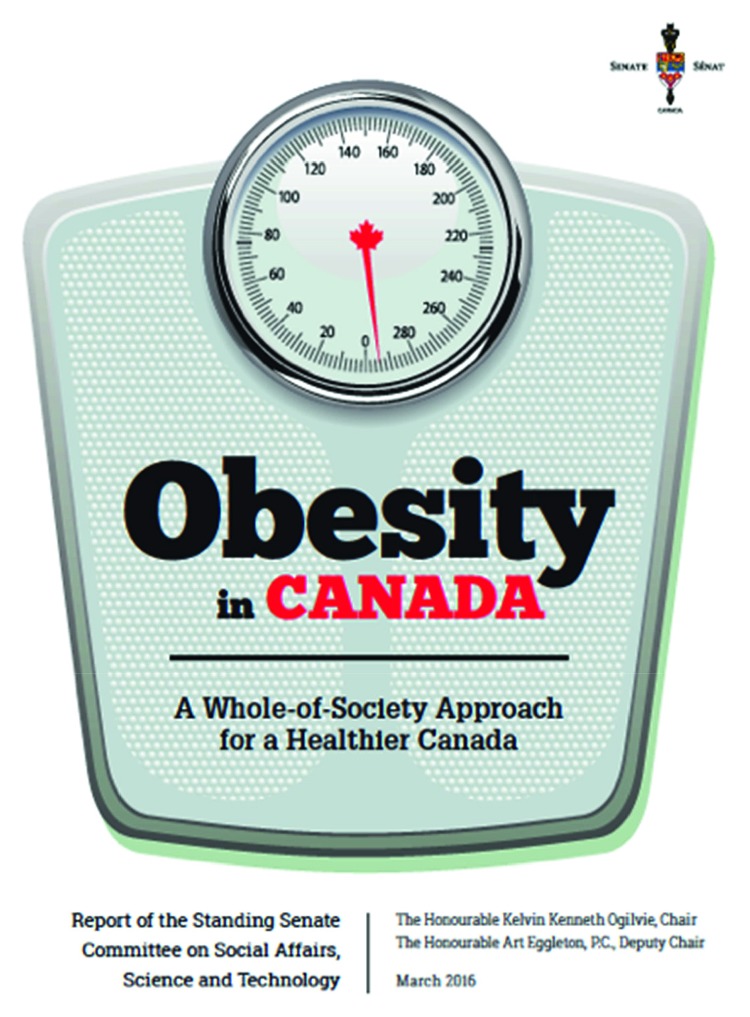
Cover of the Senate Report on Obesity in Canada [1] (reproduced with permission).

Prejudiced visual framing contributes to the weight stigma in society. The image of a weight scale adorns the cover of the Report [1] (Figure 1). This image reinforces the idea that body weight, or the number on a scale, is a proxy for health and that being above a certain weight category suggests that one is “unhealthy” or “diseased.” This is an example of the common phenomenon of negative imagery that has the potential to perpetuate weight stigma. In other contexts, visual images in news stories [39, 40] and public health campaigns aimed at obesity prevention [41, 42] have been shown to convey stigmatizing messages. One American study showed that 72% of images used in obesity-related news stories during a 2-week period in September 2009 were stigmatizing, in that the pictures only showed the lower body or abdomen of individuals with obesity, with unhealthy foods or drinks [43]. It has been argued that utilizing stigmatizing visual and linguistic stereotypical portrayals of obesity and weight-related disorders in the media is unethical and should be stopped so as not to further entrench weight stigma in its viewers [40]. From that point of view, and despite the problematic image on the cover, the Report should be commended for including a nonstereotypical image of a person living with obesity pursuing a healthy behavior such as running on a treadmill [1], p. 33. Future reports should consider including more images portraying body diversity such as this one.

## 3. Suggested Recommendations: The Report Does Not Acknowledge the Pervasiveness and Negative Consequences of Weight Stigma, but Future Reports Could

Although the Report states, “Every Canadian is affected in some way by the obesity crisis” ([1], p. iv), it does not mention that those living with obesity experience weight stigma. Despite substantive evidence on the presence and consequences of weight stigma and the work of Canadian organizations in addressing weight stigma in Canada (e.g., Canadian Obesity Network, BalancedView), the Report does not address weight stigma nor include any steps for its reduction in its 21 concluding recommendations. Below, we offer five recommendations for how future reports can be improved within a public health framework. By promoting health, equity, and social determinants of health, future reports can align more strongly with a “Whole of Society” approach (which the Report claims to take, albeit in a limited capacity).

### 3.1. Future Reports Could Better Address Social Determinants of Health (SDH)

The Report should be commended for stating that “pursuing healthy weights should involve the supportive environment of a whole-of-society approach rather than be dismissed as a purely individual responsibility” ([1], p. 10). However, its recommendations center on the individual making better lifestyle choices like eating less and exercising more, which is a common yet problematic narrative in Canadian obesity prevention policies and strategies [44]. The focus on individualism diverts attention from the need to address underlying, deeper SDH in Canada [15]. Strong science shows that the SDH, that is, the conditions in which people live, work, learn, and socialize, directly affect health and well being, and the powerful negative effects of social exclusion and discrimination cannot be overstated [16]. Research suggests that societies that have more equitable SDH have better population health and lower rates of obesity [17]. Future reports would be improved by explicitly acknowledging and discussing SDH—including ways to improve mental and social health and reduce social exclusion and discrimination, which in turn could improve health and reduce the prevalence of chronic diseases.

### 3.2. Future Reports Could Address Equity Issues Related to People Living with Obesity

The Report states, “Other key recommendations would make it easier for Canadians to make informed decisions about their diet” ([1], p. v). We have argued elsewhere [45] that weight bias is a manifestation of social inequity whereby people are judged by their body weight and thus treated unfairly in various sectors of society. Although not discussed in the Report, there is extensive evidence of unfair treatment of people living with obesity in employment, education, healthcare, interpersonal relationships, and maternity care [4].

### 3.3. Future Reports Could Incorporate the Voices of People Living with Obesity

The Report was well intended to adopt a “Whole-of-Society-Approach,” as indicated in its title, implying that all key stakeholders should be involved in improving the health of Canadians. The WHO describes a “Whole-of-Society Approach” as “[requiring] a concerted and collaborative effort by different various government ministries, businesses and civil society to sustain essential infrastructure and mitigate impacts on the economy and the functioning of society” [46]. It is important for future reports to include more considerations of civil society, including members of the public living with obesity and nongovernmental organizations in the development and dissemination of public health reports like these. In Canada, a framework that may help facilitate the goal of better engagement of civil society is the Strategy for Patient-Oriented research (SPOR) [47], which is a coalition of federal, provincial, and territorial partners dedicated to the integration of research into care by engaging patients as partners in the inception, design, and dissemination of health-related research to identify research priorities and integrate research findings into patient care and public health policy.

### 3.4. Future Reports Could Acknowledge and Discuss the Noncontrollable Aspects of Obesity

The Report does not acknowledge that obesity has been recognized as a chronic disease by the Canadian Medical Association [48], it does discuss the increased risk of acquiring other diseases such as diabetes, cardiovascular disease and the risk of premature death associated with obesity. Less emphasis in the Report was placed on causes of obesity not controlled readily by individuals, such as genetics, poverty, mental health trauma, and environmental surroundings. Further, as noted, most recommendations focus on individuals making changes to reduce their risk of obesity. Explicit acknowledgement and discussion of noncontrollable aspects of obesity could have merit: a systematic review of weight bias reduction interventions among health professionals showed that providing information on noncontrollable aspects of obesity helps reduce negative attitudes and beliefs about obesity [49]. If future reports consider inclusion of noncontrollable aspects of obesity, it could help reduce weight stigma among its readers.

### 3.5. Future Reports Could Utilize More Respectful Terminology and Framing of Obesity

Based on the evidence provided throughout this commentary, we recommend using person-first, nonaggressive, nonalarmist terminology in future reports. We further recommend that images used illustrate diverse people with different ethnicities, races, genders, sexual orientations, and body sizes, engaging in positive health behaviors, such as physical activity (like on p. 33 of the Report [1]), balanced nutrition, happy emotional states, and interacting with others. Positive image galleries from the Canadian Obesity Network [50] and the University of Connecticut Rudd Center [51] can be used free of charge with acknowledgement to the organizations in future reports.

## 4. Conclusion

The purpose of our critique was to detail important concerns with the “Obesity in Canada: A Whole-Of-Society Approach for a Healthier Canada” report that are emblematic of the weight stigma that pervades Canadian society, and to provide recommendations for future reports. We acknowledge that this critique is limited by our inability to distinguish between the government reporting and the reports of the expert stakeholders involved; however, considering that it is a government report in the public domain, we argue that critiquing it at face value is defensible.

In summary, we suggest that health policy documents and strategies in Canada (1) explicitly acknowledge and discuss the social determinants of health instead of only promoting individual agency and thereby avoid perpetuating oversimplified prevailing narratives like “eat less, exercise more”; (2) address equity issues like unequal treatment in employment, healthcare, and education for people living with obesity; (3) incorporate the voices of people living with obesity using a patient-oriented approach; (4) recognize the many factors outside of individual control that affect the prevention, treatment, and management of obesity and; (5) utilize more respectful terminology and framing of obesity by including images that portray body diversity and engagement in healthy behaviors using sensitive terminology that does not perpetuate weight stigma. Overall, future reports, campaigns, and policies should not focus on weight as a proxy for health nor utilize stigmatizing images or terminology. Instead, a truly “Whole-of-Society” approach must incorporate health-promoting messages addressing social determinants of health in Canada and engaging civil society in a meaningful way.
